# Supplementary material for: The Impact of ETV6-NTRK3 Oncogenic Gene Fusions on Molecular and Signaling Pathway Alterations
Source: Cancers (Basel). 2023 Aug 24;15(17):4246. doi: 10.3390/cancers15174246 (PMC10486691; doi:10.3390/cancers15174246)

Extended Data S1: Original western blot images used to make western blot images in figure 1 and band quantification. A) Whole western blot images used, detected for both HA epitope tagged constructs and alpha tubulin loading control. B) The western blot lanes used, shown for the entire length of the western blot membrane. C) Quantification of western blot bands with ImageJ.

Extended Data S2: Original western blot image used to make western blot images for figure 4 and band quantification. A) Whole western blot image used, detected for both phosphorylated ERK and alpha tubulin loading control B) Quantification of western blot bands with ImageJ.

Extended Data S3: Original western blot image used to make western blot images for figure 5 and band quantification. A) Whole western blot image used, detected for both HA epitope tagged constructs and alpha tubulin loading control B) Quantification of western blot bands with ImageJ.

A) Figure 1 E whole WB images

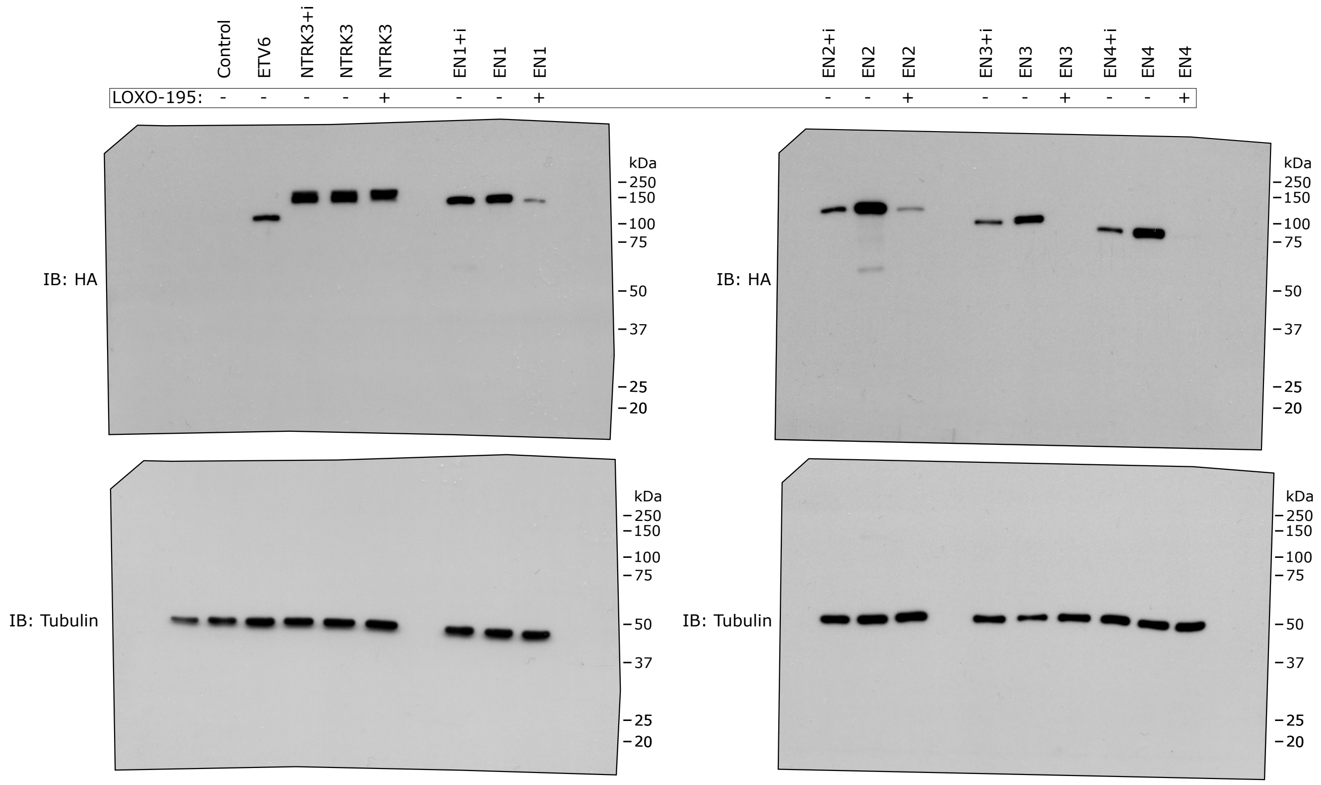

B) Figure 1 E WB bands used in figure 1

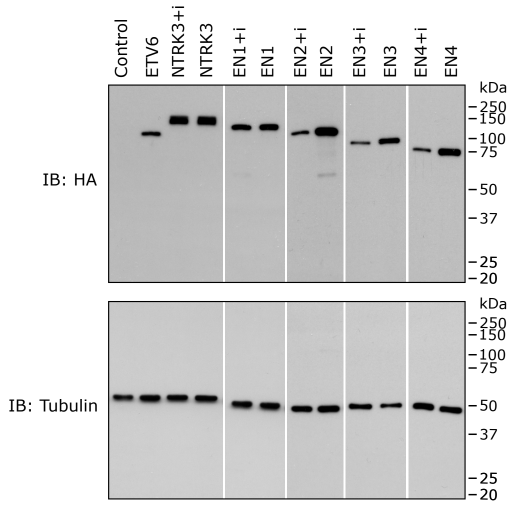

C) Figure 1 E WB band quantification

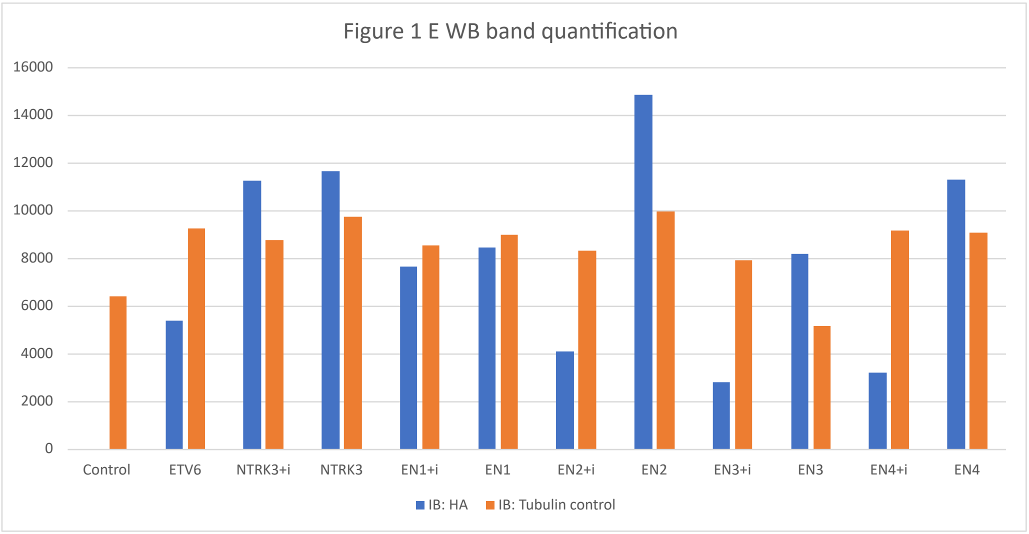

A) Figure 4 B whole WB image

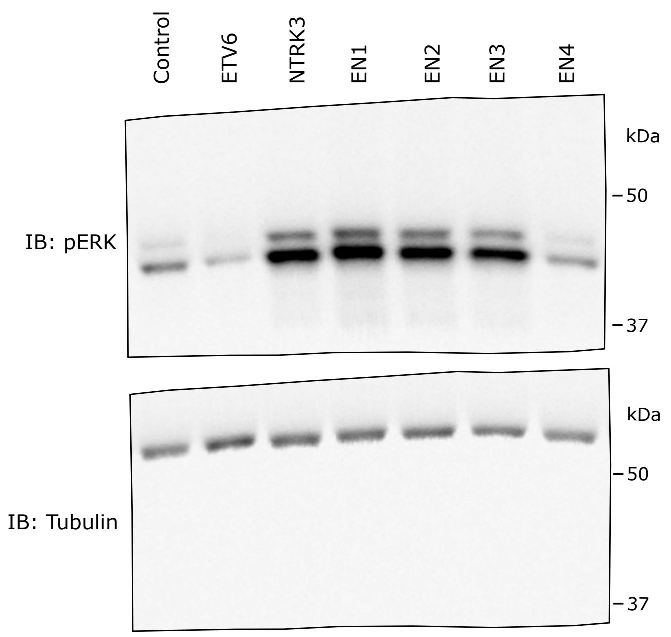

B) Figure 4 B WB band quantification

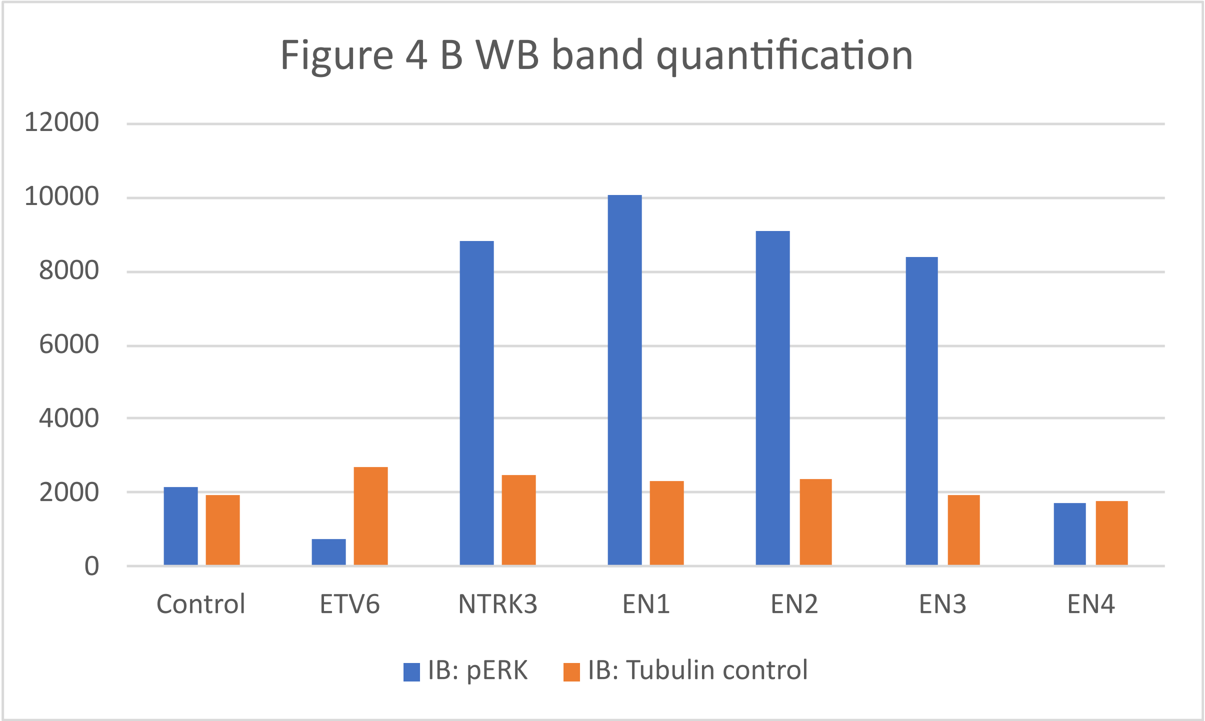

A) Figure 5 C whole WB image

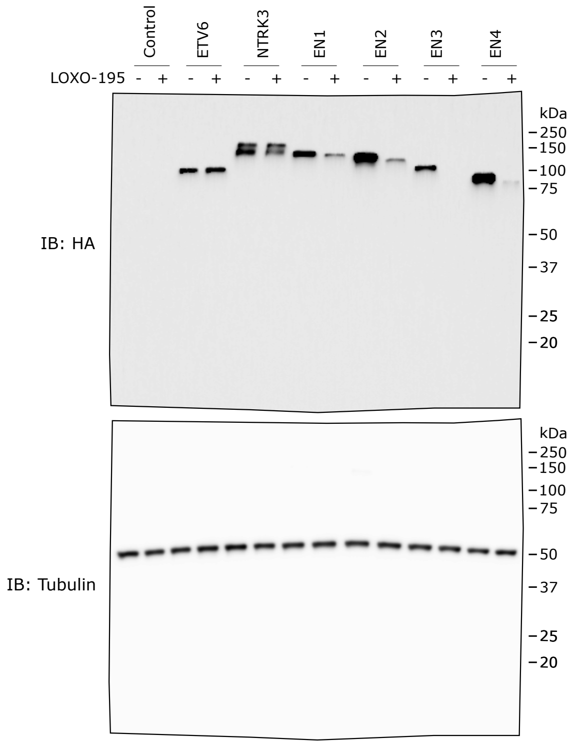

B) Figure 5 C WB band quantification

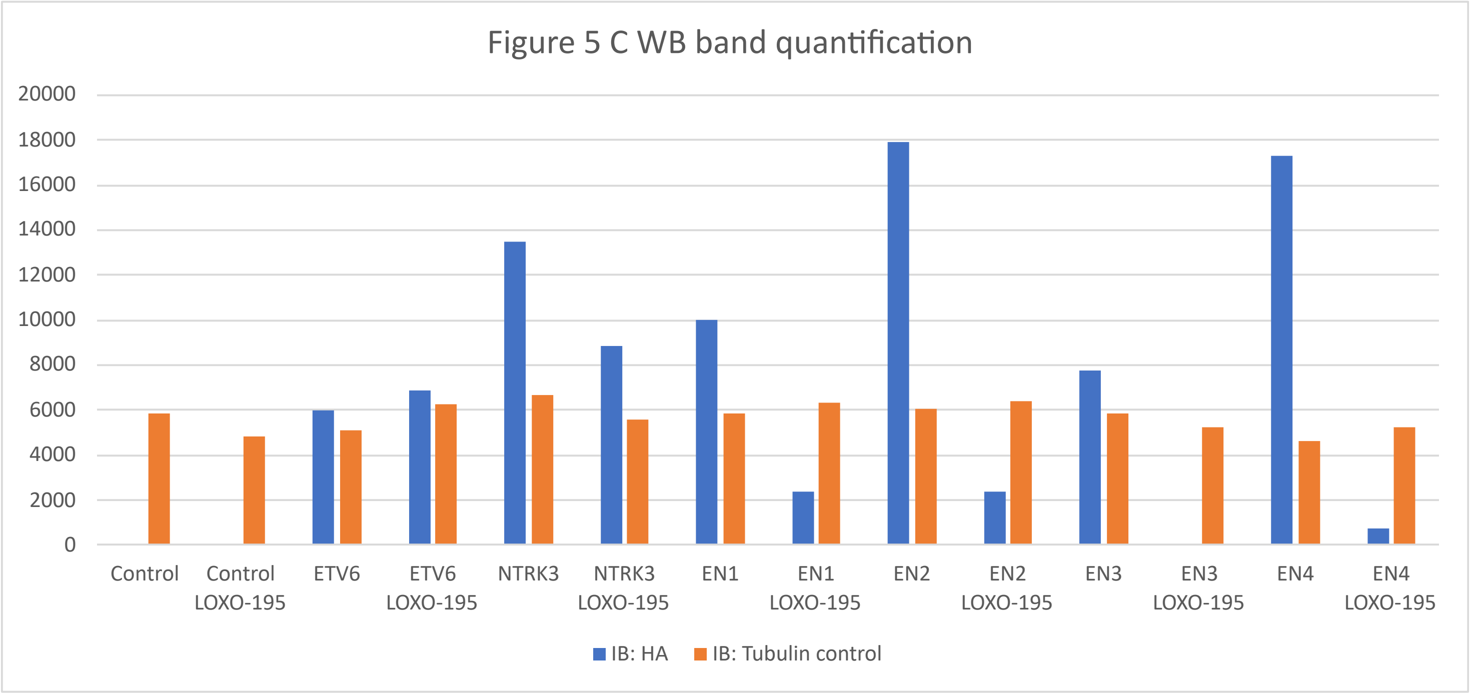

Supplement: Supplementary file 1 [file cancers-15-04246-s001.zip › Extended Data WB band quantifications.pdf]
