# Supplementary material for: The Impact of ETV6-NTRK3 Oncogenic Gene Fusions on Molecular and Signaling Pathway Alterations
Source: Cancers (Basel). 2023 Aug 24;15(17):4246. doi: 10.3390/cancers15174246 (PMC10486691; doi:10.3390/cancers15174246)
Supplement: Supplementary file 1 [file cancers-15-04246-s001.zip › Supplementary data legends.pdf]

Supplementary Figure legends

Examining the Impact of ETV6-NTRK3 Oncogenic Gene Fusions on Molecular and Signaling Pathway Alterations

Matias Kinnunen [1,2], Xiaonan Liu [1,2], Elina Niemelä [1,2], Tiina Öhman [1,2], Lisa Gawriyski [1,2], Kari Salokas [1,2], Salla Keskitalo [1,2], Markku Varjosalo [1,2].

1 Institute of Biotechnology, University of Helsinki, 00014 Helsinki, Finland
2 Helsinki Institute of Life Science, University of Helsinki, 00014 Helsinki, Finland

Supplementary Figure S1. EN and EN+i interactome comparisons.

This figure shows the analysis of clustered interactors from Figure 2 C, using reactome terms. The terms related to the overrepresented term areas are color-coded: green for cell cycle, blue for kinase signaling, and orange for Rho GTPase signaling

Supplementary Figure S2. Comparison of EN, EN2, ETV6, and NTRK3 interactors.

A) Venn diagram showing the overlap of interactors for EN1-4, EN2, ETV6, and NTRK3.

B) KEGG pathway analysis of EN1-4 unique interactors that were not found in wild type ETV6 or NTRK3.

Supplementary Figure S3. Venn diagram comparison of EN2, EN2 treated with selitrectinib, and EN2 +i interactomes.

This figure shows a Venn diagram comparing the interactomes of EN2, EN2 treated with selitrectinib, and EN2+i.
