# Supplementary material for: The Impact of ETV6-NTRK3 Oncogenic Gene Fusions on Molecular and Signaling Pathway Alterations
Source: Cancers (Basel). 2023 Aug 24;15(17):4246. doi: 10.3390/cancers15174246 (PMC10486691; doi:10.3390/cancers15174246)
Supplement: Supplementary file 1 [file cancers-15-04246-s001.zip › Supplementary Figure S1.pdf]

Cell cycle | Kinase signaling | Rho GTPase signaling

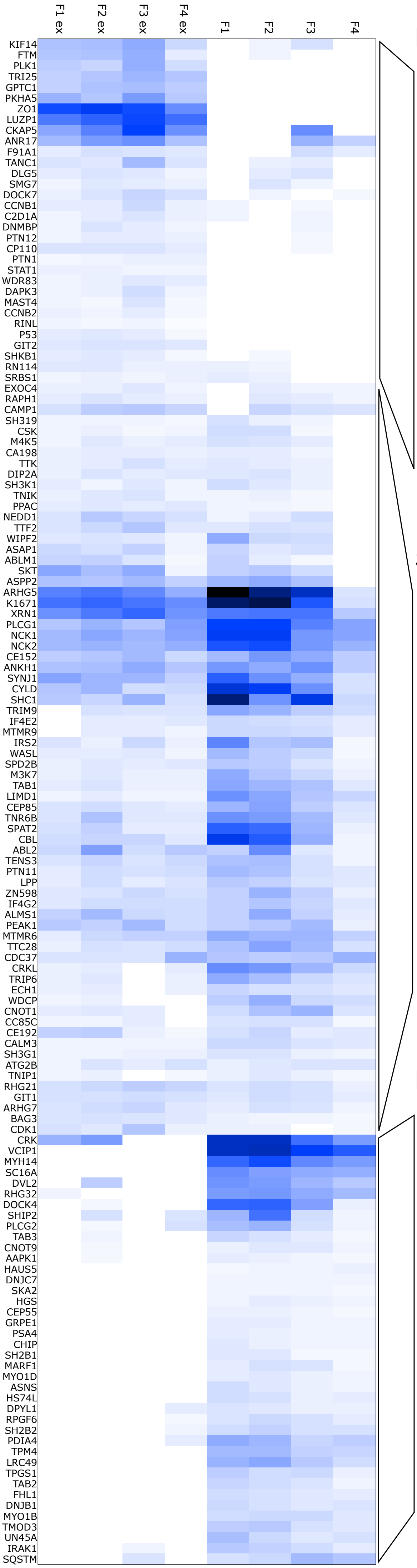

EN cluster

Pathway name	p Value
Signaling by Rho GTPases	2.89E-08
Signaling by Rho GTPases, Miro GTPases and RHOBTB3	3.74E-08
RHO GTPase cycle	1.50E-06
G2/M DNA replication checkpoint	4.57E-06
Activation of NIMA Kinases NEK9, NEK6, NEK7	2.65E-05
Mitotic Prometaphase	3.10E-05
Signaling by phosphorylated juxtamembrane, extracellular and kinase domain KIT mutants	3.24E-05
Signaling by KIT in disease	3.24E-05
Golgi Cisternae Pericentriolar Stack Reorganization	3.81E-05
Cyclin A/B1/B2 associated events during G2/M transition	4.88E-05
Resolution of Sister Chromatid Cohesion	6.42E-05
RHOV GTPase cycle	6.64E-05
Regulation of PLK1 Activity at G2/M Transition	9.59E-05
Signaling by Hippo	1.08E-04
Factors involved in megakaryocyte development and platelet production	1.14E-04
Chk1/Chk2(Cds1) mediated inactivation of Cyclin B:Cdk1 complex	1.52E-04
MET promotes cell motility	1.65E-04
Interferon alpha/beta signaling	1.67E-04
Nuclear Envelope Breakdown	1.70E-04
SARS-CoV-1 Genome Replication and Transcription	1.91E-04

Shared cluster

Pathway name	p Value
RHOV GTPase cycle	1.42E-08
RHO GTPase cycle	2.22E-06
RHOJ GTPase cycle	4.35E-06
RAC1 GTPase cycle	1.22E-05
AURKA Activation by TPX2	2.36E-05
SHC-related events triggered by IGF1R	3.94E-05
CDC42 GTPase cycle	4.46E-05
Recruitment of NuMA to mitotic centrosomes	4.65E-05
IGF1R signaling cascade	5.36E-05
Signaling by Type 1 Insulin-like Growth Factor 1 Receptor (IGF1R)	5.62E-05
RHOU GTPase cycle	9.49E-05
RHOQ GTPase cycle	1.26E-04
Signaling by Erythropoietin	2.82E-04
Anchoring of the basal body to the plasma membrane	3.81E-04
Recruitment of mitotic centrosome proteins and complexes	4.16E-04
Loss of Nlp from mitotic centrosomes	4.41E-04
Loss of proteins required for interphase microtubule organization from the centrosome	4.94E-04
InlB-mediated entry of Listeria monocytogenes into host cell	5.34E-04
Centrosome maturation	6.80E-04
Signaling by MET	7.74E-04

EN+i cluster

Pathway name	p Value
Diseases of signal transduction by growth factor receptors and second messengers	1.72E-09
Signaling by Interleukins	6.76E-07
Cytokine Signaling in Immune system	2.51E-06
Interleukin-1 family signaling	4.24E-06
Interleukin-1 signaling	5.69E-06
Constitutive Signaling by EGFRvIII	5.69E-06
Signaling by EGFRvIII in Cancer	5.69E-06
Signaling by EGFR in Cancer	6.37E-06
Signaling by PTK6	1.21E-05
Signaling by Non-Receptor Tyrosine Kinases	1.21E-05
VEGFA-VEGFR2 Pathway	1.48E-05
VEGFR2 mediated vascular permeability	1.53E-05
Signaling by VEGF	1.72E-05
Signal Transduction	2.13E-05
Signaling by Rho GTPases	2.13E-05
Signaling by Rho GTPases, Miro GTPases and RHOBTB3	2.61E-05
JNK (c-Jun kinases) phosphorylation and activation mediated by activated human TAK1	2.68E-05
activated TAK1 mediates p38 MAPK activation	3.06E-05
Signaling by ALK fusions and activated point mutants	3.91E-05
Signaling by ALK in cancer	3.91E-05
