# Supplementary figures and images for: The Impact of ETV6-NTRK3 Oncogenic Gene Fusions on Molecular and Signaling Pathway Alterations
Source: Cancers (Basel). 2023 Aug 24;15(17):4246. doi: 10.3390/cancers15174246 (PMC10486691; doi:10.3390/cancers15174246)

**A**

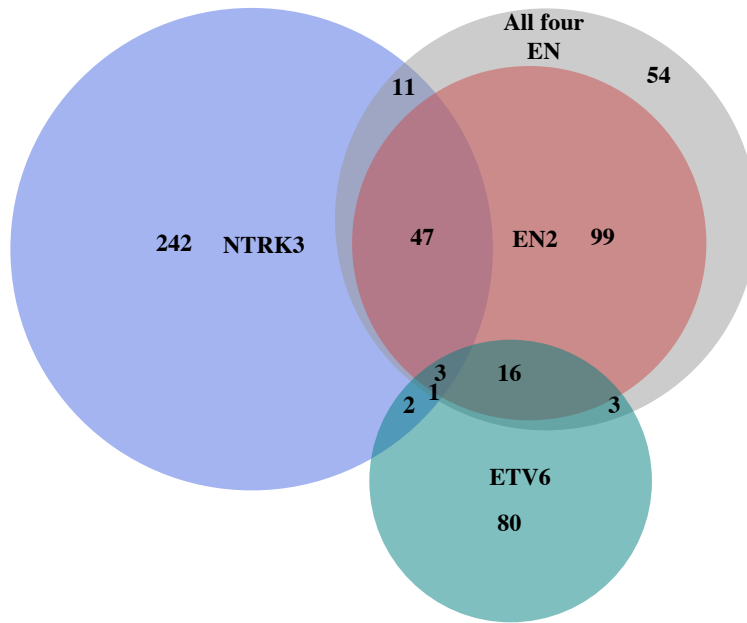

**B**

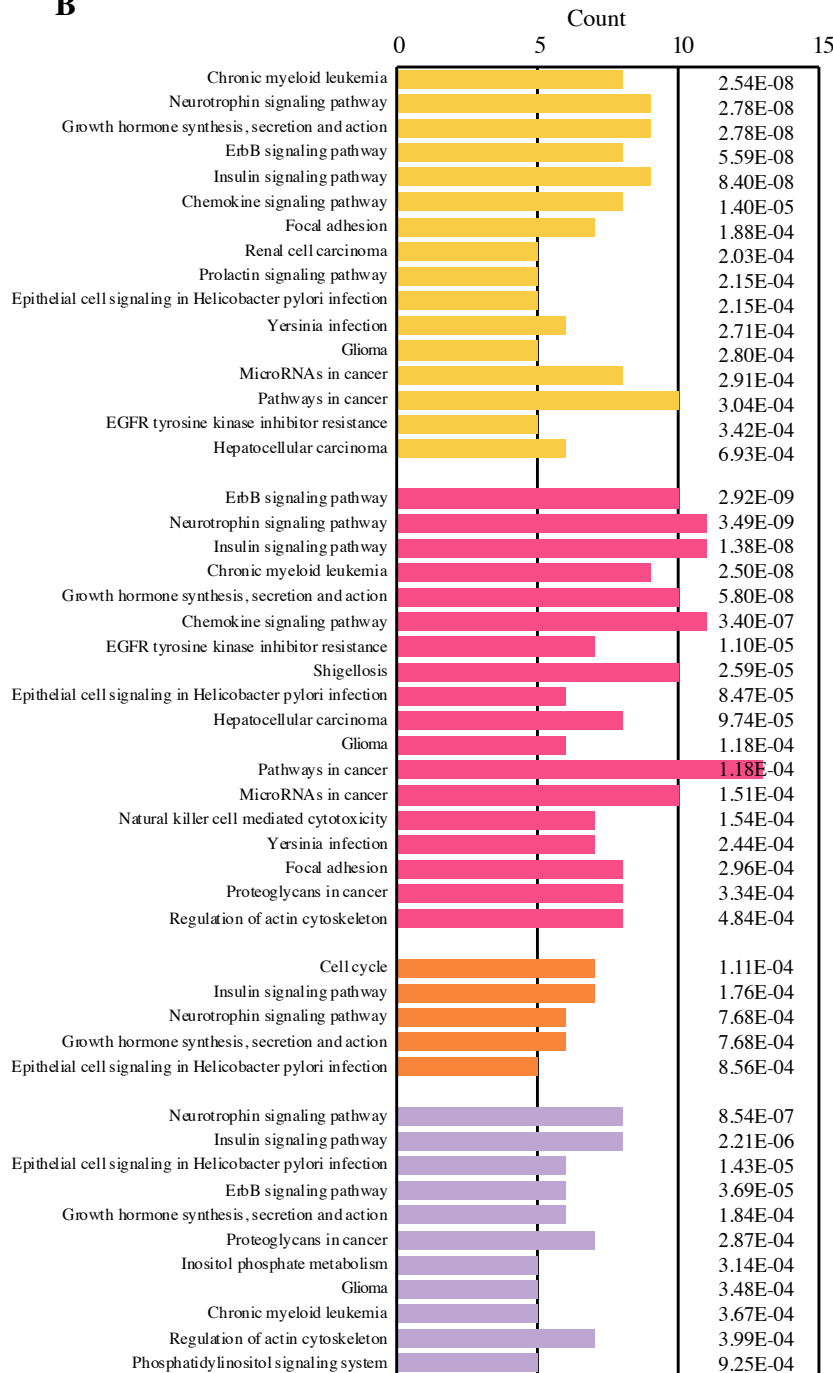

Supplement: Supplementary file 1 [file cancers-15-04246-s001.zip › Supplementary Figure S2.pdf]

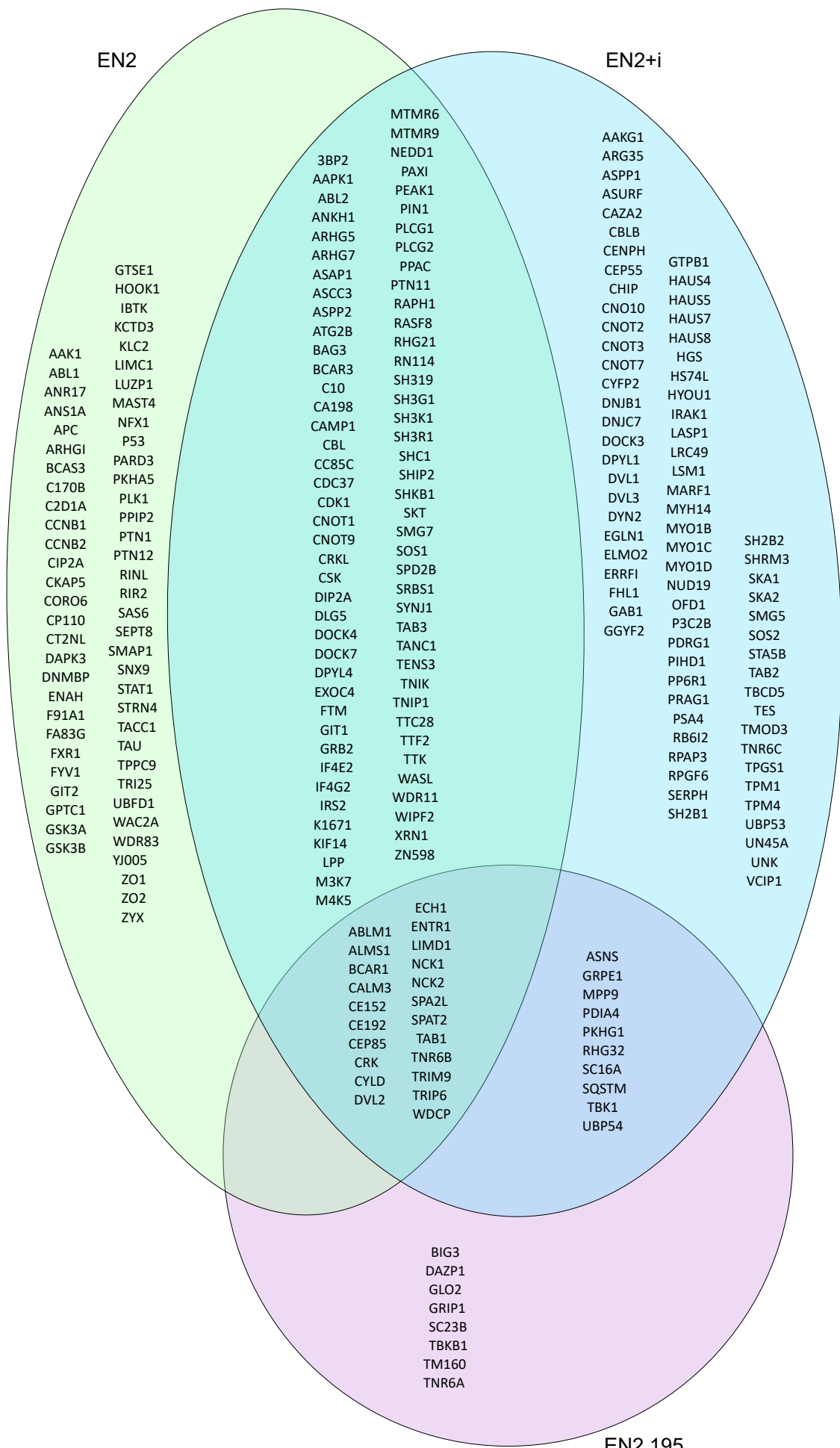

Supplement: Supplementary file 1 [file cancers-15-04246-s001.zip › Supplementary Figure S3.pdf]
